# Strategies to address inequity of uncorrected refractive error in the Western Pacific: A modified Delphi process

**DOI:** 10.1111/opo.13348

**Published:** 2024-06-16

**Authors:** Ian McCormick, Kelvin Tong, Nurliyana Abdullah, Carmen Abesamis-Dischoso, Theresa Gende, Effendy Bin Hashim, S. May Ho, Isabelle Jalbert, Belmerio Jeronimo, Elenoa Matoto-Raikabakaba, Koichi Ono, Prabhath Nishantha Piyasena, Jaymie T. Rogers, John Szetu, Minh Anh Tran, Dennis Yan-yin Tse, Ye Win, Tiong Peng Yap, Sangchul Yoon, Mayinuer Yusufu, Matthew J. Burton, Jacqueline Ramke, Promoting Equity in Refractive Error Services in the Western Pacific Study Group

**Affiliations:** 1 International Centre for Eye Health, London School of Hygiene and Tropical Medicine, London, UK; 2 Li Ka Shing Faculty of Medicine, The University of Hong Kong, Pok Fu Lam, Hong Kong; 3 PAPRSB Institute of Health Sciences, Universiti Brunei Darussalam, Bandar Seri Begawan, Brunei Darussalam; 4 Eccles Institute of Neuroscience, The John Curtin School of Medical Research, Australian National University, Canberra, Australian Capital Territory, Australia; 5 The Professional ODs Society, Pasig, Philippines; 6 Divine Word University, Madang, Papua New Guinea; 7 The Fred Hollows Foundation New Zealand, Madang, Papua New Guinea; 8 Department of Eye and Vision Science, Institute of Life Course and Medical Sciences, University of Liverpool, Liverpool, UK; 9 Ministry of Health, Putrajaya, Malaysia; 10 The Fred Hollows Foundation, Melbourne, Victoria, Australia; 11 School of Optometry and Vision Science, UNSW Sydney, Sydney, New South Wales, Australia; 12 Department of Ophthalmology, Guido Valadares National Hospital, Dili, Timor-Leste; 13 Colonial War Memorial Hospital, Suva, Fiji; 14 Department of Ophthalmology, Juntendo University Faculty of Medicine, Tokyo, Japan; 15 Directorate of Policy Analysis and Development, Ministry of Health, Colombo, Sri Lanka; 16 Centre for Public Health, School of Medicine, Dentistry and Biomedical Sciences, Queen’s University Belfast, Belfast, UK; 17 School of Optometry and Vision Science, University of Auckland, Auckland, New Zealand; 18 The Fred Hollows Foundation New Zealand Regional Eye Centre, Honiara, Solomon Islands; 19 Department of Ophthalmology and Optometry, Hanoi Medical University, Hanoi, Vietnam; 20 School of Optometry, The Hong Kong Polytechnic University, Hong Kong, China; 21 Sight for All, Yangon, Myanmar; 22 IGARD Vision Therapy Centre, Singapore City, Singapore; 23 Department of Medical Humanities and Social Sciences, College of Medicine, Yonsei University, Seoul, South Korea; 24 Department of Surgery (Ophthalmology), The University of Melbourne, Melbourne, Victoria, Australia; 25 Centre for Eye Research Australia, Royal Victorian Eye and Ear Hospital, East Melbourne, Victoria, Australia; 26 National Institute for Health Research Biomedical Research Centre for Ophthalmology at Moorfields Eye Hospital NHS Foundation Trust and UCL Institute of Ophthalmology, London, UK

**Keywords:** access, equity, financial protection, refraction, refractive error, spectacles

## Abstract

**Purpose:**

Uncorrected refractive error is the leading cause of vision impairment globally; however, little attention has been given to equity and access to services. This study aimed to identify and prioritise: (1) strategies to address inequity of access to refractive error services and (2) population groups to target with these strategies in five sub-regions within the Western Pacific.

**Methods:**

We invited eye care professionals to complete a two-round online prioritisation process. In round 1, panellists nominated population groups least able to access refractive error services, and strategies to improve access. Responses were summarised and presented in round 2, where panellists ranked the groups (by extent of difficulty and size) and strategies (in terms of reach, acceptability, sustainability, feasibility and equity). Groups and strategies were scored according to their rank within each sub-region.

**Results:**

Seventy five people from 17 countries completed both rounds (55% women). Regional differences were evident. Indigenous peoples were a priority group for improving access in Australasia and Southeast Asia, while East Asia identified refugees and Oceania identified rural/remote people. Across the five sub-regions, reducing out-of-pocket costs was a commonly prioritised strategy for refraction and spectacles. Australasia prioritised improving cultural safety, East Asia prioritised strengthening school eye health programmes and Oceania and Southeast Asia prioritised outreach to rural areas.

**Conclusion:**

These results provide policy-makers, researchers and funders with a starting point for context-specific actions to improve access to refractive error services, particularly among underserved population groups who may be left behind in existing private sector-dominated models of care.

## Introduction

Uncorrected refractive error is the leading cause of moderate or severe distance vision impairment and the second most common cause of blindness globally.^1^ It affects people across their life course, with negative consequences on education, productivity and quality of life.^2–5^

The importance of uncorrected refractive error as a cause of vision impairment was reinforced by the adoption by the World Health Assembly of effective refractive error coverage (eREC) as one of two indicators to monitor global eye health.^6^ eREC describes the proportion of the population in need of refractive error correction that has had that need met (i.e., people whose unaided vision in the better eye is worse than 6/12 but can see 6/12 or better with available correction).^7^ There is currently substantial interest in eREC as an indicator that can contribute to monitoring Universal Health Coverage (UHC), and it will be considered for inclusion when the UHC Index^8^ is revisited by the World Health Organisation (WHO) in 2024.

Fortunately, most refractive error can be corrected with spectacles which are a non-invasive, cost-effective treatment.^9^ However, in contrast to services for other causes of vision impairment such as cataract—which requires secondary care and is generally available to some extent at government facilities—refractive error services are largely provided in primary care settings by the private sector.^9^ Spectacles are often perceived as cosmetic rather than medical devices and, in some settings, optical companies (including opticians and manufacturers) may deprioritise investment in services among lower-income or remote populations to ensure viability (i.e., profitability).^10–13^ Therefore, in settings without sufficient levels of financial protection, some population groups will be left underserved. An example of an underserved group is women, who tend to experience uncorrected refractive error disproportionately compared to men, and this gender disparity tends to be worse among older than younger age groups and in countries with lower Human Development Index scores.^14^ Another example is older people in rural areas of lower-income countries.^4^

There are regional differences in the prevalence of distance refractive error globally, which is highlighted within the Western Pacific region. For example, myopia has increased to ‘epidemic’ levels in East and Southeast Asia in recent years, while its prevalence is predicted to remain relatively low in Oceania over the coming decades.^15,16^ While the prevalence and magnitude of refractive error varies by region, the proportion that remains uncorrected also varies. Countries with a high prevalence of myopia and good access to refractive error services may have a low proportion of people with uncorrected refractive error (i.e., ‘unmet need’ for optical correction). Conversely, in countries with a low prevalence of refractive error, there may still be a high proportion of people with an unmet need for correction if access to refractive error services is insufficient or unaffordable. Separately to distance refractive error, presbyopia affects all adults from around 40 years of age in all world regions; however, the magnitude of near vision impairment caused by uncorrected presbyopia also varies by region, largely associated with the accessibility and affordability of optical correction.^9^

In 2021, Member States at the 74th World Health Assembly endorsed a global target to increase eREC for distance and near vision by 40 percentage points, with increases in all relevant population subgroups.^17^ To increase eREC, countries need to improve access to services and financial risk protection (i.e., safeguarding against financial hardship imposed by paying for [refractive] care^18^) for underserved groups, but evidence for how to achieve this equitably is lacking. While refractive error does not exist in isolation from other eye conditions, we wished to address it directly in response to the new global target. Therefore, an online Delphi-like prioritisation study was performed to collate a range of expert opinions in the region.

The aim of this project was to identify and prioritise: (1) strategies to address the inequity of access to refractive error services (refraction and spectacle correction) and (2) population groups to target with these strategies in five sub-regions of the Western Pacific region (the Global Burden of Diseases regions of Southeast Asia, East Asia, Oceania, Australasia and high-income Asia Pacific regions).

## Methods

This project used a modified Delphi-like prioritisation process and is reported according to the relevant items in the Delphi-specific guidance.^19^ Ethics approval for this study was received from the London School of Hygiene & Tropical Medicine Ethics Committee (Ref 25715) and all panellists provided informed consent to participate.

### Panellist selection

We used purposive sampling to select panellists, with a focus on ophthalmic professionals with knowledge and experience of refractive error services, including optometrists, ophthalmologists, ophthalmic nurses and experts in public health for eye care. We aimed to recruit 15–20 panel-lists from each of the five selected sub-regions (Southeast Asia, East Asia, Oceania, Australasia and high-income Asia Pacific), with equal numbers of men and women. The panellists were identified via personal networks and snowballing from existing relationships, including via recommendations from the World Council of Optometry.

### Data collection

Data collection involved two rounds of an online process using Qualtrics software (qualtrics.com) and drew on a similar process for cataracts.^20^ The questionnaire was provided in English and Chinese.

In round 1, panellists provided information about their gender, years of eye care experience, country and main field of experience. They were then asked four open-ended questions about the situation in their country, and encouraged to give answers with as much detail as possible:

1. Which population subgroups experience the most difficulty in accessing refractive error services?
2. For those in the most vulnerable population subgroups (i.e., those identified in question 1) with refractive error, what are the most effective strategies that can increase access to distance vision refraction services (i.e., getting a prescription)?
3. For those in the most vulnerable population subgroups with refractive error, what are the most effective strategies that improve access to distance vision correction (i.e., getting spectacles)?
4. For those in the most vulnerable population subgroups, what are the most effective strategies that improve access to near vision (presbyopic) screening and correction?

When panellists nominated a group that was an intersection of two or more population groups, for example, ‘rural women’, these were separated and presented as individual groups. For the nominated strategies, content analysis was used to identify the major themes for each of the three main service types: (1) distance vision refraction, (2) distance vision correction (i.e., getting spectacles) and (3) near vision screening and correction. All ideas generated in round 1 were de-duplicated and included in round 2, with no further items added.

In round 2, panellists ranked each population subgroup identified in round 1 on a 5-point scale in terms of: (1) the extent of difficulty the group experienced in accessing refractive error services in their setting (from the least/no difficulty [0] to the most/extreme difficulty [5]) and (2) the size of the group experiencing difficulty accessing refractive error services when they needed it (from the smallest [0] to the largest group [5]). For each group, a ‘not applicable’ item was available if the panellist felt a group was not relevant in their setting.

To prioritise strategies to improve access to refractive error services, panellists were presented with the list of nominated strategies from round 1 and asked 12 questions across 5 themes—reach, acceptability, sustainability, feasibility and equity (Box 1). Panellists selected and ranked the top three strategies (1 being the highest) in response to each of the 12 questions below. We randomised the order in which the strategies and themes were presented to prevent bias.

### Data analysis

Analysis was completed separately for each of the five sub-regions and an average (mean) across all regions was calculated.

For the most promising strategies for each of our three main service types, the top-ranked choice from each panellist for each question (Box 1) was allocated 3 points, the second choice 2 points and the third choice 1 point. The points each strategy received in each question were summed, and then the scores of all questions in each category of reach, acceptability, sustainability, feasibility and equity were summed. The total score of each category was divided by the number of questions in the category to yield an adjusted score that gave equal weight to each of the five categories (e.g., the equity category had three questions, so the total equity score was divided by 3). The sum of the adjusted scores of all five categories was then calculated to find the strategy with the highest score overall. To compare the scores across the five regions, each regional score was divided by the number of panellists in that region to give a comparable panellist-weighted average score.

### Situational analysis of panellist countries

After online data collection was performed, we contacted one panellist per country included in the study to collect basic information about the status of optometry, for example, the cadre principally responsible for refraction, the number of practitioners and the scope of practice of optometrists (if relevant).^21^ We also asked them about financial protection for refractive error services in their setting. We questioned whether national health finance pooling mechanisms pay for (1) refraction and (2) spectacles for everyone, or one or more population sub-groups.

## Results

### Characteristics of panellists

In total, 75 of 84 invited panellists completed both rounds (89% participation rate); 55% of panellists were female (*n* = 41; Table 1). Panellists were primarily clinicians (*n* = 44, 59%) but also included researchers and decision-makers; more than half (58%) had at least 20 years of eye care experience. Southeast Asia had the highest number of panel-lists (*n* = 20) and Oceania the fewest (*n* = 11).

### Population subgroups most unable to access services

After we de-duplicated round 1 responses, we had 15 distinct population subgroups considered least able to access refractive error services. Across all sub-regions, the groups considered to have most difficulty were people without housing, refugees and people with low socio-economic positions. The groups considered to be largest of those who may experience difficulty were people with low socioeconomic positions, people living in remote/rural areas and the elderly. When the mean value was calculated across the two criteria, the prioritised groups across all subregions were people with low socioeconomic positions, people living in remote/rural areas and people without housing. Women were considered the group with the least difficulty accessing refractive services in all sub-regions except Oceania, and when all sub-regions were combined (Table 2).

There were differences across the five sub-regions. The greatest consensus within a sub-region was seen for refugees experiencing difficulty in East Asia (score = 4.67), rural and remote people experiencing difficulty (4.55) and being a large group (4.36) in Oceania, people with low social support experiencing difficulty in high-income Asia Pacific (4.33) and Indigenous peoples experiencing difficulty in Australasia (4.29).

### Strategies to improve access to distance refraction services

Round 1 generated 211 suggestions for strategies to improve access to distance vision refraction services. We de-duplicated and consolidated these responses into 15 separate strategies put forward in round 2. Each subregion prioritised a different strategy as the most promising (highest score in Table 3). When all sub-regions were combined, the strategies considered most promising were to *organise regular vision screening services to identify refractive error* (considered most promising in East Asia), to *reduce out-of-pocket costs by providing publicly funded refraction services to underserved groups* (considered most promising in Australasia) and to *implement mobile outreach to rural and remote areas* (considered most promising in Oceania). *Training community- or mid-level health workers* was the second most promising strategy in Oceania while being a relatively low priority in other sub-regions. Australasia considered *improving cultural safety* and *establishing permanent services in underserved areas* much more promising compared to other regions while placing much lower emphasis on *strengthening school eye health programmes* compared to other regions. High-income Asia Pacific was the only sub-region to have *raised awareness by providing health education/promotion* ranked in the top three strategies.

### Strategies to improve access to distance correction services

In round 1, panellists provided 156 responses for strategies to improve access to distance vision correction services, which were de-duplicated and consolidated into 11 separate strategies in round 2 (Table 4). The most promising strategies to improve access to distance vision correction were fairly similar across the five sub-regions. Two of the three highest-ranked strategies across all sub-regions combined involved *reducing out-of-pocket costs*, either by *providing publicly funded distance prescription spectacles to certain groups* (which was considered the most promising in Australasia and the highest-rated strategy overall) or via *public–private partnerships* (considered most promising in Southeast Asia). *Raising awareness and acceptance of spectacle wear* was also in the top three when all sub-regions were combined while being the most promising in high-income Asia Pacific and East Asia. The strategies considered most promising in Oceania were slightly different, where *establishing dispensing services within government eye clinics* and *improving logistics of spectacle frame and lens supply* featured alongside reducing out-of-pocket costs. *Including spectacles in health insurance schemes* ranked behind public funding for certain groups and public–private partnerships as strategies to reduce out-of-pocket costs in all sub-regions except East Asia, where it ranked above public–private partnerships (Table 4).

### Strategies to improve access to near vision screening and correction services

Round 1 saw 162 responses for strategies to improve access to near vision screening and correction services, which were consolidated into 10 unique strategies in round 2 (Table 5). When the five sub-regions were combined, the strategies considered by panellists to be most promising were to *integrate services with community activities where older adults gather* (considered most promising in Southeast Asia), to *reduce out-of-pocket costs by providing publicly funded near prescription spectacles to certain groups* (considered most promising in Australasia) and to *train community-level members or health workers to conduct screening and provide readymade presbyopic spectacles* (considered most promising in East Asia and Oceania). *Raising awareness* was considered most promising in high-income Asia Pacific, mirroring this sub-region’s result for distance vision correction (Table 5). The use of *readymade spectacles when suitable* was ranked fifth overall, while second in high-income Asia Pacific and third in Oceania. Another highly rated strategy in some regions was to *introduce a policy to recommend regular screening in the presbyopic age group*, which was ranked second in both Australasia and East Asia but considerably lower in other sub-regions.

### Technology

While technological innovation was mentioned as a strategy to improve access to refraction and its correction in Round 1, it was not prioritised in Round 2, with development of low-cost mobile refraction equipment and innovation in spectacle frame manufacturing ranking near or at the bottom.

### Financial protection

Among the 17 countries represented by panellists in this study, financial protection for refraction was much more likely than for spectacles. Key informants from 11 (65%) countries (Australia, Brunei Darussalam, China, Fiji, Japan, Malaysia, Myanmar, Solomon Islands, South Korea, Sri Lanka and Timor-Leste) reported a mechanism for health finance pooling for refraction that covered all citizens (Table 6). Three further settings had financial protection for refraction for one or more population subgroups: for people with vision impairment (Hong Kong, Papua New Guinea and New Zealand [only based on diagnosis of conditions such as high myopia or keratoconus]), for children (Papua New Guinea and New Zealand [only children whose parents have a community services card]) and for the elderly (Papua New Guinea), leaving Philippines, Singapore and Vietnam as the countries informants reported to be without financial protection for refraction for anyone. In contrast to widespread support for refraction, financial protection against the cost of spectacles was limited across the region. No country provided financial protection for spectacles for everyone, and seven countries provided at least partial support for at least one population subgroup. People with low income were the group most commonly targeted (five countries), while children (three countries), people with vision impairment (four countries) and the elderly (two countries) were also targeted. This left 10 settings (Brunei Darussalam, China, Fiji, Hong Kong, Malaysia, Philippines, Papua New Guinea, Singapore, Timor-Leste and Vietnam) in which informants reported no financial protection for spectacles was available for anyone from national health finance pooling.

## Discussion

To meet the World Health Assembly’s global target of an equitable 40 percentage-point increase in effective refractive error coverage by 2030, access to refractive error services must be improved among all population groups.^22^ We assembled a panel of 75 experienced stakeholders from 17 countries across the Western Pacific region to identify and prioritise the most promising strategies to improve access to refractive error services, as well as the population groups to target with these strategies.

The population groups prioritised by all panellists were people with low socioeconomic positions, people living in rural/remote locations and people without housing. Regional differences were evident, with groups considered the highest priority in some sub-regions—such as Indigenous peoples in Australasia and Southeast Asia—obtaining a relatively low priority in other sub-regions. A survey in Australia identified worse refractive error coverage in Indigenous Australians compared to non-Indigenous Australians^23^; however, there is little evidence to support monitoring efforts to increase eREC equitably in the region. Gender is a commonly captured equity dimension in population-based eye health surveys, while more eREC data disaggregated by ethnicity, place of residence and socioeconomic position will be required to monitor the subgroups prioritised here. In addition, regular health equity impact assessment of refractive error policy-making may help to monitor progress towards UHC.^10^

In response to the World Health Assembly resolution to increase eREC and promote equity, governments and their partners are looking for evidence on how this can be achieved. The sub-regional differences observed in proposed priorities suggest a need for context-specific solutions.^24^ For example, panellists in Oceania prioritised strategies to address remoteness and the issues with spectacle supply chain and dispensing infrastructure in their sub-region, which differed markedly from strategies selected by panellists from other settings. Furthermore, panellists in Australasia were more inclined than other panellists to prioritise improving cultural safety and health literacy of eye health services, reflecting the need to address structural racism of health services for Indigenous populations in this sub-region.^25^ For all sub-regions except Oceania, improving physical access to refractive error services—in terms of increasing outreach services or the number or distribution of personnel—was deemed a much lower concern than improving affordability.

Increasing public funding was among the most prioritised strategies across the region to improve access to each refraction, spectacles and near vision correction, reinforcing the call made by Fardow et al.^26^ that (uncorrected) refractive error should be considered a health care need and addressed as a public health duty to avoid the inequity produced when health care is commercialised.^10^ The high priority for financial protection as a strategy awarded by panellists reflects the mixed picture across the region, particularly in relation to public funding to support people to access spectacles (shown in Table 6). Given that cost is an important barrier to accessing spectacles for some groups,^27^ countries wishing to avoid perpetuating inequity in efforts to increase eREC may need to revisit the extent to which financial protection can be extended to certain population groups, and how that fits within broader national health financing priorities.

The magnitude of the need for refractive error correction across all world regions means both public and private sector solutions are required to increase eREC equitably, and public–private partnerships were recognised as a priority strategy in several sub-regions. The importance of the private sector to achieve UHC and the need for better public–private engagement was reinforced by WHO in 2020.^28^ In its *Strategy Report on the Governance of the Private Sector for Universal Health Coverage*, WHO emphasised the need for the private sector (for profit and not for profit) to align better with government agenda and for governments to provide a strong regulatory environment around these issues. Changes to professional and/or optical market governance or regulation were, however, not nominated as promising strategies by any of our panellists, despite panellists working in countries that sit along a spectrum of regulation, ranging from countries without regulation (Myanmar, Solomon Islands and Vietnam) through to those that are highly regulated (Australia, Japan and New Zealand).

There is strong support from global organisations for countries to make major changes to the structure of refractive error care. In its ‘2030 In Sight’ strategy, the International Agency for the Prevention of Blindness called for market changes to expand equitable access to eye care and for the private sector to share data with governments on refractions and optical devices sold so that progress can be monitored.^29^ In addition, in 2024, WHO launched the ‘SPECS 2030’ initiative which emphasises the role of multi-sector engagement, legislation and regulation in scaling up access to spectacles.^30^

Another potential theme for the SPECS 2030 agenda is expansion of the refractive error service workforce. The provider-to-population ratio for optometrists/refractionists varied greatly across the countries represented by our panellists. Despite this variation, increasing the number of optometrists/refractionists or dispensing opticians was not a priority for improving access to refraction or spectacles in any of the sub-regions. The only workforce-related strategy to be prioritised for distance refractive error was training non-specialists to conduct refraction in Oceania. Training community and primary health workers to screen and provide presbyopic correction was a more popular strategy and is aligned with a new WHO-endorsed open online programme called Training in Assistive Products.^31^ The lack of emphasis on human resources for refraction may reflect the high proportion of panellists who were clinicians or may also reflect a perception that availability of personnel is much less of an issue than affordability in the region. In pursuit of the 2030 global target to improve eREC, in most contexts, it is likely to be counter-productive in the longer term to consider refractive error services in isolation from primary eye care. Across the region, training eye care workers who provide refraction to uniform levels of proficiency could be an aspiration to support integrated people-centred eye care through enhanced disease detection and management at the primary care level.

Given the dearth of evidence on how to improve access to refractive error services, we are encouraged to see that SPECS 2030 will include a research programme and we particularly look forward to the health systems and policy research that will emerge. Reflecting on the priorities from the exercise presented here, strategies to improve eREC will vary considerably by region, but key areas for attention might include building the evidence base for including refraction and/or spectacle provision in essential health benefits packages for all or some population sub-groups. On the demand side, priorities for exploration may include developing strategies for demand generation, including awareness of refractive error, presbyopia and reducing stigma about spectacle wear.^32–34^ A platform for shared learning—such as on the sustainability of different models of spectacle provision—would be a valuable component of the SPECS 2030 initiative.

In addition to improving access, efforts to increase eREC must also ensure that refractive error services are of good quality. One approach that has been trialled in the region assesses the quality of refractive error care (Q.REC), which summarises service characteristics and determines whether prescribed spectacles are within acceptable tolerance ranges.^35^ We did not seek suggestions on strategies to improve and maintain quality of refractive error services in this study. Should this exercise be undertaken, we recommend the quality framework adopted by WHO be used, as recently demonstrated in a review of strategies to strengthen quality of cataract services.^36^

These results must be interpreted in the context of several limitations. First, we were unable to recruit panellists from all countries in the region, so some contexts are not represented in the results. All panel exercises reflect the perspectives of participants recruited; a different mix of clinicians to researchers or programmatic personnel may have prioritised different strategies. Furthermore, aggregating results to the regional level can obscure differences between countries within the region. An example is the priority given to providing publicly funded refraction services to underserved groups in Australasia—given that this strategy is available in Australia (albeit imperfectly for rural dwellers), this regional result was likely to be dominated by strong consensus among panellists from New Zealand, where the absence of such strategies for the vast majority of the population is a recognised issue.^37^ Second, in considering underserved groups, we did not disaggregate to account for intersectionality, for example, rural women, who likely face cumulative barriers to access, as found for cataract services.^38^ In some regions, we may have masked poorer access for females by presenting women as a single population group. Third, we gave equal weight to each of the five themes when calculating the overall priority scores per strategy, which may not reflect the weight panellists would place on each of the themes—panellists may have different weighting across the five themes in Box 1 which were not reflected in the final results.

In pursuit of universal health coverage in eye health, access to refractive error services must be improved among all population groups. The strong consensus from our panellists highlights that in the Western Pacific region, financing of refractive error care is a critical issue, with a recognised need for public funding to ensure equitable access. With the ambitious global target to increase eREC by 2030, there is an urgent need for evidence-informed action on how access and quality of refractive error care can be increased equitably.

## Figures and Tables

**Table 1:** Characteristics of panellists who completed both rounds of the study.

Panellist characteristics	*n*	(%)
*Sex*		
Female	41	55
Male	34	45
*Sub-region*		
Southeast Asia	20	27
Australasia	15	20
East Asia	15	20
High-income Asia Pacific	14	19
Oceania	11	14
*Main field of experience*		
Clinician/Practitioner	44	59
Clinical research	7	9
Eye health service research	7	9
Management/Leadership	7	9
Epidemiology	5	8
Government/Ministry of Health	4	5
International NGO	1	1
*Eye care experience (years)*		
<10	9	12
10–19	23	30
20–29	21	28
30 or more	23	30
Total	75	100

**Table 2:** Population groups most unable to access refractive error services by sub-region and regional average, arranged by the highest five-region mean values across the two criteria (mean not shown in table).

	Australasia		High-income Asia Pacific		Southeast Asia		East Asia		Oceania		Five-region average	
Priority groups	Most difficulty	Biggest group	Most difficulty	Biggest group	Most difficulty	Biggest group	Most difficulty	Biggest group	Most difficulty	Biggest group	Most difficulty	Biggest group
People with low socioeconomic position	4	3.92	3.31	3.08	4.25	3.6	3	3.23	4.18	4.09	3.75	3.58
People living in remote/rural areas	3.71	3.21	2.75	2.43	3.9	3.1	3.58	3.54	4.55	4.36	3.7	3.33
People without housing	4.21	3	4.27	2.5	3.81	3.38	3.58	3.67	3.63	2.57	3.9	3.02
People with low social support (e.g., no family to accompany)	3.64	2.71	4.33	3.38	3.7	2.8	3.08	3.38	3.91	3.09	3.73	3.07
Elderly	3.15	3.15	3.23	3.23	3.6	2.9	2.69	2.69	3.91	3.64	3.32	3.12
People with disability (non-vision related, e.g., low mobility)	3.54	2.36	3.62	2.92	3.95	2.6	2.92	2.62	3.91	3.18	3.59	2.74
People with low health literacy	3.64	3.29	3.46	2.92	3.1	2.7	3.08	3.08	3.3	2.82	3.32	2.96
Refugees/Asylum seekers	3.67	2.54	3.6	2.5	4.14	2.36	4.67	3	3	1.67	3.82	2.41
People living in institutional facilities (elderly care homes and prisons)	3.08	2.64	3.69	3	3.47	2.2	3.15	3.15	3.56	2.7	3.39	2.74
Indigenous populations	4.29	3.79	2.25	2.2	4.25	2.31	2.4	1.71	3.29	2.75	3.3	2.55
Children	2.86	2.54	1.82	1.83	3.1	2.8	2.2	2	3.73	3.36	2.74	2.51
Non-dominant ethnic minorities	3.38	3.15	3.13	2.13	3.21	2.25	2.2	1.92	2.5	2	2.88	2.29
Migrants	2.92	2.54	2.89	2.25	3.69	2.57	1.9	1.64	2.83	2	2.85	2.2
People who do not speak the dominant language	3.31	2.54	2.4	2.3	2.67	1.94	2.91	2.08	2.22	2	2.7	2.17
Women	1.67	2.67	1.63	1.55	2.21	2.45	1.73	1.93	3.27	3.27	2.1	2.37

*Note*: Red and green represent the high and low ends of the scale, from most/extreme to least/no difficulty and from largest to smallest group, respectively.

**Table 3:** Strategies to improve access to distance vision refraction services (i.e., getting a prescription) by sub-region, arranged by highest regional average score.

Strategies to improve access to distance vision refraction services (i.e., getting a prescription)	Sub-regional priorities					
	Australasia	High-income Asia Pacific	Southeast Asia	East Asia	Oceania	Regional average
Organise regular vision screening services to identify refractive error (e.g., national programme for preschool children and free annual check for elderly)	24.8	47.4	34.0	**48.0**	14.5	33.7
Reduce out-of-pocket costs by providing publicly funded refraction services to underserved groups (e.g., elderly, low-income groups and children)	**51.6**	24.2	21.1	26.7	34.6	31.7
Implement mobile outreach to rural and remote areas (with appropriate refraction equipment for quality service provision)	23.7	20.6	34.0	17.8	**55.8**	30.4
Integrate refraction services with primary care and other allied health services (e.g., better referral system with General Medical Practitioners and co-locate with pharmacy/other health services)	28.1	30.4	**41.3**	22.8	17.5	28.0
Raise awareness by providing health education/promotion on availability of services and need for and benefits of refractive error services	16.7	**48.0**	19.7	29.2	15.8	25.9
Strengthen school eye health programmes—ensure regular and equitable coverage of regions, population groups and age groups	5.4	25.4	30.9	33.5	20.2	23.1
Target services to underserved population groups (e.g., women, Indigenous peoples, refugees, high deprivation, prisons and home visits for older people)	28.7	22.6	23.5	22.8	16.4	22.8
Train community health workers/community nurses/mid-level personnel to conduct refraction	14.0	7.8	8.5	18.2	55.1	20.7
Establish more permanent refraction services in underserved areas and incentivise optometrists/refractionists to work there	33.8	16.0	19.1	12.3	17.3	19.7
Provide transport to assist people to reach refraction services	18.0	13.2	11.9	12.3	8.9	12.9
Reduce out-of-pocket costs by including refraction in health insurance schemes	10.8	10.4	11.0	18.6	5.5	11.3
Increase the number of optometrists/refractionists with skills to provide comprehensive, quality care	3.2	8.8	14.8	11.8	17.3	11.2
Develop easy-to-use, low-cost, mobile auto-refraction equipment	6.3	4.6	11.6	15.7	15.0	10.6
Improve cultural safety and health literacy of eye care services (e.g., interpreter services, delivery where people feel comfortable and diverse workforce with competence in cultural safety)	29.8	11.5	4.9	3.5	3.0	10.5
Establish policy on provision of refraction within occupational health requirements	5.2	9.0	13.9	6.7	3.2	7.6

*Note*: Green and red represent the highest and lowest prioritised strategies, respectively. The highest-ranked strategy of each sub-region is bolded.

**Table 4:** Strategies to improve access to distance vision correction by sub-region, arranged by highest regional average score.

Strategies to improve access to distance vision correction (i.e., spectacles)	Sub-regional priorities					
	Australasia	High-income Asia Pacific	Southeast Asia	East Asia	Oceania	Regional average
Reduce out-of-pocket costs by providing publicly funded distance prescription spectacles to certain groups (e.g., elderly, low-income groups and children)	**87.7**	50.4	40.6	49.7	58.8	57.4
Raise awareness and acceptance of spectacle wear by providing health education/promotion on symptoms of uncorrected refractive error, availability of services and benefits of correction	46.2	**66.7**	44.8	**61.4**	28.6	49.5
Reduce out-of-pocket costs by providing distance spectacles via public–private partnerships, including industry corporate social responsibility programmes, charitable funds and NGOs	40.1	49.0	**56.4**	25.8	54.8	45.2
Establish spectacle dispensing services (including glazing laboratories) within public/government eye clinics	28.2	31.7	34.7	35.9	**61.6**	38.4
Reduce out-of-pocket costs by including distance prescription spectacles in health insurance schemes	19.9	30.8	25.6	35.7	14.2	25.3
Establish policies for workplaces and schools that encourage the provision and use of spectacles where required	24.0	22.3	38.1	16.8	6.1	21.5
Improve the logistics of spectacle frame and lens supply (e.g., improved availability of stock and reduced delivery time)	15.2	10.8	18.6	12.7	31.8	17.8
Increase the number of dispensing opticians and/or optical lab technician graduates with skills to fulfil simple and complex spectacle prescriptions	6.7	13.9	14.5	29.8	22.7	17.5
Collect population-based data on distance spectacle needs, including among population subgroups	19.3	20.6	12.5	10.3	6.6	13.9
Procure appropriate spectacle frame designs for the population (in terms of fit and cosmetic appeal)	7.1	2.2	11.6	13.3	11.0	9.0
Leverage technological innovation in 3D printing to increase access to spectacles	5.6	1.3	2.7	8.5	3.8	4.4

*Note*: Green and red represent the highest and lowest prioritised strategies, respectively. The highest-ranked strategy of each sub-region is bolded. Abbreviations: NGO, non-governmental organisation; 3D, three-dimensional.

**Table 5:** Strategies to improve access to near vision screening and correction services by sub-region, arranged by highest regional average score.

Strategies to improve access to near vision screening and correction services	Sub-regional priorities					
	Australasia	High-income Asia Pacific	Southeast Asia	East Asia	Oceania	Regional average
Integrate screening services and correction with community activities where older adults gather (e.g., religious or cultural events)	36.3	48.7	**54.1**	38.1	64.2	48.3
Reduce out-of-pocket costs by providing publicly funded near-prescription spectacles to certain groups (e.g., elderly and low-income groups)	**61.5**	36.0	34.5	39.9	42.9	43.0
Train community members/village health workers/community nurses to conduct screening and provide readymade presbyopic spectacles	24.5	31.9	34.8	**52.3**	**66.4**	42.0
Raise awareness by providing health education/promotion on symptoms of near vision impairment, availability of services and benefits of correction	39.4	**55.1**	37.0	34.1	31.0	39.3
Use readymade spectacles when suitable (including messaging around importance of regular eye checks)	39.2	49.2	31.4	30.9	44.3	39.0
Introduce a policy to recommend regular screening in the presbyopic age group (e.g., during medical examination in the workplace)	50.0	34.2	33.0	42.9	7.8	33.6
Reduce out-of-pocket costs by providing near spectacles via public–private partnerships, including industry corporate social responsibility programmes, charitable funds and NGOs	24.2	28.4	41.6	12.7	32.9	28.0
Create a social enterprise model with the purpose of providing readymade reading glasses to underserved groups (e.g., women and refugees)	6.1	11.2	18.4	14.1	8.3	11.6
Use new technologies such as artificial intelligence and mobile health applications to screen for presbyopia	12.3	3.5	13.7	16.9	2.2	9.7
Offer monovision as an option when undergoing cataract surgery	6.5	1.9	1.5	17.7	0.0	5.5

*Note*: Green and red represent the highest and lowest prioritised strategies, respectively. The highest-ranked strategy of each sub-region is bolded. Abbreviation: NGO, non-governmental organisation.

**Table 6:** Indicative overview of refractive error services in countries represented in this study, as reported by a panellist from each country.

Sub-region	Country^a^	Main refracting profession	Optometry scope of practice^b^ (highest level)	Refractionist-to-population ratio (per 10,000)	Does national health finance pooling mechanisms pay for refraction for					Does national health finance pooling mechanisms pay for spectacles for				
					All	Subgroups of the population				All	Subgroups of the population			
						Vision impaired	Children	Elderly	Low income		Vision impaired	Children	Elderly	Low income
High-income Asia Pacific	Japan	Ophthalmologist/Orthoptist	2	1.71	Yes	Yes	Yes	Yes	Yes	No	Yes	Partial	No	Yes
Southeast Asia	Sri Lanka	Optometrist/Ophthalmic technician	2	0.30	Yes	Yes	Yes	Yes	Yes	No	No	Yes	Partial	Yes
Australasia	Australia	Optometrist	4	2.24	Yes	Yes	Yes	Yes	Yes	No	Partial	Partial	Partial	Yes
High-income Asia Pacific	South Korea	Optometric optician	2	4.32	Yes	Yes	Yes	Yes	Yes	No	Yes	No	No	No
Southeast Asia	Myanmar	Ophthalmologist/Refractionist	4	0.04	Yes	Yes	Yes	Yes	Yes	No	Yes	No	No	No
Oceania	Solomon Islands	Ophthalmologist/Ophthalmic nurse	0	0.11	Yes	Yes	Yes	Yes	Yes	No	No	Yes	No	Yes
East Asia	China	Optometrist	2	0.04	Yes	Yes	Yes	Yes	Yes	No	No	No	No	No
High-income Asia Pacific	Brunei Darussalam	Optometrist	2	0.80	Yes	Yes	Yes	Yes	Yes	No	No	No	No	No
Oceania	Fiji	Optometrist/Ophthalmic nurse	3	0.17	Yes	Yes	Yes	Yes	Yes	No	No	No	No	No
Southeast Asia	Malaysia	Optometrist/Optician	2	1.44	Yes	Yes	Yes	Yes	Yes	No	No	No	No	No
Southeast Asia	Timor-Leste	Optometrist	0	0.23	Yes	Yes	Yes	Yes	Yes	No	No	No	No	No
East Asia	Hong Kong SAR	Optometrist	3	3.00	No	Yes	No	No	No	No	No	No	No	No
Australasia	New Zealand	Optometrist	4	1.71	No	Partial	Partial	No	Partial	No	Partial	Partial	No	Partial
Oceania	Papua New Guinea	Other	0	0.002	No	No	No	No	No	No	No	No	No	No
Southeast Asia	Philippines	Optometrist	3	0.44	No	No	No	No	No	No	No	No	No	No
Southeast Asia	Vietnam	Mixed	3	0.20	No	No	No	No	No	No	No	No	No	No
High-income Asia Pacific	Singapore	Optometrist/Optician	3	4.44	No	No	No	No	No	No	No	No	No	No

*Note*: Settings where health care vouchers are available for certain groups but are not specific to refraction or spectacles were recorded as a ‘no’ response as other healthcare services may be prioritised for voucher use. Table cell colours used to visually represent whether national health finance pooling is available per population group (Yes=green, Partial=amber, No=red).

a Hong Kong Special Administrative Region (SAR) of China reported separately.

b 0 = No scope of practice; 1 = Optical technology services: Management and dispensing of ophthalmic lenses, ophthalmic frames and other ophthalmic devices that correct defects of the visual system; 2 = Visual function services: Optical technology services plus investigation, examination, measurement, recognition and correction/management of defects of the visual system; 3 = Ocular diagnostic services: Optical technology services plus visual function services plus investigation, examination and evaluation of the eye and adnexa, and associated systemic factors to detect, diagnose and manage disease; 4 = Ocular therapeutic services: Optical technology services plus visual function services plus ocular diagnostic services plus use of pharmaceutical agents and other procedures to manage ocular conditions/disease.

## Box 1 Questions presented for strategies in Round 2

**A Reach**

- Which strategy would reach the *largest amount of population?*
- Which strategy would reach the *population most in need?*

**B Acceptability**

- Which strategy would be most acceptable to people with *uncorrected refractive error*?
- Which strategy would be most acceptable to *local government agencies* (e.g., Ministry of Health)?
- Which strategy would be most acceptable to frontline *health workers*?
- Which strategy would be most acceptable to *commercial optical providers*?

**C Sustainability**

- Which strategy is likely to have an *immediate* impact?
- Which strategy is likely to be most *sustainable* in the long term?

**D Feasibility**

- Which strategy will be the most *feasible* to implement in your setting?

**E Equity**

- Which strategy is the most effective in improving access for *elderly*?
- Which strategy is the most effective in improving access for *people with low socioeconomic positions*?
- Which strategy is the most effective in improving access for *people living in rural/remote areas*?

## Key points

- There is an ambitious 2030 global target for effective refractive error coverage, but no evidence on how to achieve this equitably; this study begins to fill the evidence gap in the Western Pacific region.
- Given that refractive error services are predominantly provided in the private sector, the increase in public funding identified as a priority to address inequity highlights the importance of effective public–private partnerships.
- Beyond the strong consensus identified for public funding, the diverse range of strategies prioritised across sub-regions emphasises the need for context-specific approaches to promote equity in access to refractive error services.
